# The Impact of Myopia Control Spectacle Lens Designs on Visual Function

**DOI:** 10.1007/s44402-026-00026-2

**Published:** 2026-03-05

**Authors:** Alfredo Desiato, Hiu Yan Lam, Reena Rani Anand, Inderjit Chatha, Nicola S. Logan, Amy L. Sheppard, James S. Wolffsohn, Deborah Laughton, Leon N. Davies

**Affiliations:** 1 Optometry & Vision Science Research Group, School of Optometry, Aston University, Birmingham, UK; 2 SightGlass Vision Inc, Dallas, Texas USA

**Keywords:** Accommodative facility, Accommodative response, Contrast sensitivity, Myopia control, Reading performance, Visual acuity, Visual performance

## Abstract

**Purpose:**

To profile the immediate effect of defocus-modulating and contrast-modulating myopia control spectacle lens interventions on visual function.

**Methods:**

Healthy myopic (mean spherical equivalent (MSE) −4.25 to −0.50 D) young adults, corrected with contact lenses, wore diffusion optics technology (DOT), defocus incorporated multiple segments (DIMS), highly aspherical lenslets (HAL) and standard single vision (SV) plano trial spectacle lenses, in a prospective, single-visit, double-blind, four-way randomised crossover study. Distance and near high- and low-contrast visual acuity (VA), contrast sensitivity, reading performance, accommodative facility, visual search task and accommodative accuracy were assessed foveally through the central zone (CZ) and/or peripheral zone (PZ) of the lenses.

**Results:**

Twenty participants (16 female) were recruited, with a mean (±SD) age of 22.4 (±2.72) years and MSE −2.21 (±1.10) D. VAs through the PZ differed significantly between myopia control lenses and SV, with the HAL and DIMS exhibiting lower VA across all testing conditions (all *p* <0.05) and DOT demonstrating equivalence to SV with high contrast letters. Contrast sensitivity was similarly reduced for all lenses through the CZ, while HAL and DIMS performed worse than both SV and DOT through the PZ (*p* <0.01). Near acuity threshold, reading speed and critical print size through the PZ were comparable for SV, DOT and HAL (all *p* >0.05), whereas DIMS exhibited worse near acuity threshold and critical print size (*p* <0.001). No significant differences emerged for error score (*p* = 0.53), accommodative facility refocusing cycles (all *p* >0.05) or visual search duration (CZ: *p* = 0.68; PZ: *p* = 0.35). Accommodative response was similar across lenses (all *p* >0.05); however, SV had lower variability at distance through the PZ than DOT, HAL and DIMS (*p* <0.001).

**Conclusion:**

All three myopia control lenses exhibited visual performance comparable to standard SV lenses through the CZ. Clinicians should note differences in visual performance, especially VA and reading speed through the lens periphery and their relative testing.

Key-Points

- The use of myopia control spectacle lenses has minimal effect on visual performance, whether the design relies on defocus modulation or contrast modulation.
- Myopia control spectacle lenses provide visual outcomes comparable to standard lenses, with differences that might relate to individual wearer preferences.
- Assessing visual acuity, particularly through the lens periphery, appears to be the most indicative clinical test and should be included in the evaluation of myopia control spectacle lenses.

## Introduction

Myopia affects around a third of children and adolescents worldwide [1], and its prevalence is expected to increase to 50% of the global population by 2050 [2]. This is particularly concerning due to the link between axial length increase and the risk of visual impairment from retinal pathology [3]. Nonetheless, practitioners in some countries can now rely on several optical intervention options to control refractive error and eye length progression [4]. Among these, myopia control spectacle lenses have shown excellent safety and efficacy in randomised controlled trials [5–7], and have gained rapid popularity as a first-choice myopia control treatment around the world [8].

The scientific basis of myopia control spectacle lens designs originates from the role of the peripheral retina in controlling localised eye growth, independent of cortical control [9]. These designs typically consist of a central zone (CZ) incorporating the distance refractive correction, as in standard single-vision (SV) lenses, surrounded by a treatment zone containing lenslets—either spherical [9] or aspherical [10]—of relative positive optical power to induce myopic defocus or microscopic diffusers to modulate retinal image contrast [11]. While an increasing body of evidence confirms the ability of these designs to control myopia [5–7], it has been suggested that the presence of the treatment zone in the periphery may impact visual performance [12].

Multiple studies on myopia control spectacle lenses have reported minimal or transient effects on visual function when viewing through the CZ [9, 13–15]. Defocus incorporated multiple segments (DIMS) lenses have been reported to exhibit comparable performance with respect to conventional SV control in children’s vision and heterophoria at distance and near, amplitude and lag of accommodation and stereopsis [9, 14]. Similarly, no clinically relevant impact of highly aspherical lenslet (HAL) lenses was found when assessing children after 1 year of wear compared to SV lenses for the same vision attributes [13]. More recently, the visual performance of diffusion optics technology (DOT) lenses, which modulate retinal image contrast, was assessed against SV lenses, demonstrating comparable outcomes in terms of visual acuity (VA), contrast sensitivity, reading metrics and stereopsis [15], as well as accommodative response [16] in children.

However, the evaluation of visual functions beyond the conditions and methodologies routinely employed in optometric practice (e.g., photopic high contrast VA in primary gaze) has been indicated to quantify the potential impact on real-world visual performance associated with myopia control lens designs, with significant differences in VA observed under reduced contrast and lighting conditions [13]. In fact, high and low contrast VA when viewing through the lens periphery has been reported to be reduced for both HAL [17, 18] and DIMS [19, 20] lenses.

Thus, considering that the foveal image in primary gaze can be influenced by both paracentral and peripheral rays [15], and that visual performances through the lens periphery may vary depending on contrast and lighting levels, a more comprehensive assessment of visual performance may provide a more accurate real-world representation of children’s visual experience [17]. Additionally, this would enable practitioners to distinguish better the contributions of different lens designs to vision on an individual level. Therefore, additional clinical elements appear to be necessary in the evaluation of the designs of myopia control spectacle lenses [12].

Moreover, independent of their safety and efficacy, the clinical outcomes of myopia control lenses may be affected by a reduced level of adherence [21], which gains particular relevance when considering that increased myopia control efficacy has been reported in full-time wearers [22], with further evidence of dose-dependent effects [23]. Hence, as reduced visual performance has been associated with a higher risk of non-adherence to optical correction [24], it becomes important to assess the impact of myopia control lenses on visual performance. Hitherto, most study designs have compared myopia control lens performance against SV controls, and more rarely, including alternative versions of the same design or across similar designs. Thus, a more comprehensive understanding of the potential influences of myopia control lens designs on visual performance, assessed by direct comparison across designs based on different principles, will enable eye care practitioners to offer more extensive assistance in selecting the most suitable options for an individual child.

Hence, the aim of this study was to evaluate the visual performance of myopia control spectacle lens designs. An extended battery of tests was used in young adults to compare the visual outcomes of defocus-modulating and contrast-modulating technologies with those of standard SV lenses.

## Methods

### Study Design

This prospective, randomised, non-dispensing, double-masked, cross-over trial adhered to the tenets of the Declaration of Helsinki and was given a favourable opinion by the Health and Life Sciences Research Ethics Committee of Aston University (HLS21094). Participant inclusion parameters were between 18 and 30 years of age, best corrected VA of at least 0.00 logMAR, with a mean spherical equivalent (MSE) of at least −0.50 D, astigmatism <1.00 DC and anisometropia ≤2.00 D by non-cycloplegic open-field autorefraction (WAM-5500, Grand Seiko, grandseiko.com) [25]. Exclusion criteria included: pregnancy, aphakia or pseudophakia, binocular vision problems (e.g., amblyopia, strabismus or nystagmus), current or evolving ocular pathology, previous ocular surgery, systemic conditions and/or treatment potentially having an influence on vision or visual function and previous or current myopia control treatment. Eligible participants were enrolled for baseline screening after providing written informed consent to participate.

A 20-participant sample was targeted as, at 80% power and a familywise significance level of *p* <0.008 (Bonferroni correction for six pairwise comparisons), this would be sufficient to detect differences of: 0.03 logMAR in VA (SD = 0.03 logMAR) [26]; 0.08 D in accommodative lag (SD = 0.09 D) [25]; 10 words per minute and 0.15 logMAR in critical print size for reading metrics (SD = 11.5 words per minute; 0.17 logMAR) [27] and 0.14 cycles per degree in contrast sensitivity (SD = 0.16 units) [28].

During a single study visit, participants were rendered functionally emmetropic by wearing a suitably powered spherical daily-wear contact lens (stenfilcon A, CooperVision, coopervision.com) and subsequently wore four plano trial spectacle lenses in a randomised order: DOT (SightGlass Vision Inc., sightglassvision.com), HAL (Essilor, essilor.com) and DIMS (Hoya, hoyavision.com) lenses, with single vision lenses serving as the control condition.

Each plano test lens was glazed into a full-aperture trial lens (38 mm diameter). All measurements were taken from the right eye only, immediately after the test lens was placed into a trial frame for the measurement. Throughout the assessments, a trial lens occluder was used to cover the left eye to prevent vergence from influencing the results. The interpupillary distance was measured and the trial frame was aligned accordingly. The back vertex distance of all lenses was set to 12 mm. Testing room illumination was calibrated to 330 lx for photopic and 10 lx for mesopic conditions.

To evaluate the differential influence of lens portions on visual performance as a function of eye gaze position, measurements were taken during foveal fixation through the CZ and peripheral zone (PZ) of all test lenses. For measurements through the CZ, the lenses were mounted with their optical centres aligned with the pupil centre in the primary gaze position within the trial lens housing. For measurements through the PZ, the setup was adjusted to account for differences among the myopia control lens designs. Since the CZ diameter varies (5 mm for DOT lenses and 9 mm for HAL and DIMS lenses), the lenses were decentred by 10 mm horizontally and the central 10 mm of each lens was masked using a circular opaque black sticker of the corresponding size. Additionally, due to variations present in the size and shape of the treatment areas, the more peripheral regions (corresponding to the non-treatment peripheral portion of the DIMS lenses) were covered with opaque black tape across all lens types, to ensure that performance was assessed exclusively through the treatment areas [29].

### Visual Acuity

VA was assessed using a crowded logMAR Sloan letter (Early Treatment of Diabetic Retinopathy Study format) chart (Precision Vision, precision-vision.com) at distance (4 m) and near (40 cm) at both high (96%) and low (10%) Michelson contrast levels. Letter-by-letter scoring (0.02 logMAR) and the three errors termination rule were employed [30].

### Contrast Sensitivity Function Testing

The ability to map the complete contrast sensitivity function provides a more comprehensive assessment of visual function, rather than relying solely on data from discrete spatial frequencies or contrast levels [28]. Thus, a mobile app-based contrast sensitivity test, displayed on a tablet computer (iPad, apple.com), was used to assess the complete contrast sensitivity function [28]. The observer used a stylus to indicate where they could see the extent of a sinusoidal grating, which varied in spatial frequency along the *x*-axis and contrast along the *y*-axis. The test was first performed under photopic conditions (with and without a glare source) and then under mesopic conditions (without a glare source) after 10 min of light adaptation. All assessments were performed at a fixed viewing distance of 40 cm, approximating the use of portable digital devices, including scenarios with reduced lighting. For each condition, the testing procedure was repeated at least three times until high consistency was achieved (software real-time analysis). The glare source consisted of strip lights attached to either side of the tablet, emitting 115,000 cd/m² at an angle of ±12.3°. Before data collection, participants performed a practice trial without any testing lens in place to familiarise themselves with the test.

### Reading Metrics

Maximum reading speed, critical print size and near VA threshold were assessed at a distance of 40 cm using a digital version of an extended MNRead test [28, 31], with measurements taken exclusively through the PZ for all test lenses. Randomised short paragraphs, progressively decreasing in size, were displayed on an electronic tablet and participants were instructed to read the sentences aloud as quickly and accurately as possible. Any missing/incorrect words for each print size were manually recorded by the examiner based on analysis of the voice recording. A reading speed curve (reading speed versus print size) was plotted for each testing condition to determine the maximum reading speed (the plateau of the supra-threshold reading speed), the critical print size (the smallest print size that yielded 90% of the maximum reading speed) and the near VA threshold (the smallest print size that could be read).

### Accommodative Facility

Participants, positioned with a chin and head rest, were instructed to view sequentially a distant target and then shift their gaze downward to view a near target (40 cm), to obtain foveal viewing through the CZ for the distant target and the PZ for the near target, with lenses unmasked. The targets consisted of adapted distance and near Hart Chart tests, comprising 10 rows of characters (each containing 8 letters and 2 numbers), randomised between presentations, corresponding to 0.20 logMAR VA for both distances [32]. The number of refocusing cycles completed between the distance and near targets within a 1-min period was recorded, along with the number of errors. Prior to data collection, participants completed a shortened version of the task as a practice trial to ensure familiarity with the procedure.

### Visual Search Task

The Circles Search Test is a visual search task designed to require serial processing [33]. Adapted versions of the charts consisted of 15 rows of shapes, each containing a randomly located circle among six ellipses of randomised orientations (0°, 45°, 90° and 135°), with a major axis of 12 mm and a minor axis of 10 mm. Four different versions of the test were printed on plain white paper and mounted on a table stand positioned perpendicular to the participant, with the test administered at 40 cm. Participants were required to identify the target circle in each row as quickly as possible by marking a dot in its centre with a permanent marker. The examiner used a digital stopwatch to time the visual search task, recording both response time and error scores. A practice trial was provided for participants to gain familiarity with the test before data collection commenced. This task was conducted separately through the CZ and PZ, following the masking procedures described earlier.

### Accommodative Response and Variability

Monocular accommodation stimulus–response measures were taken using an open-field autorefractor (WAM-5500, Grand Seiko, grandseiko.com) [25]. Using a 5 Hz sampling rate, ~100 refraction readings were averaged over a duration of 20 s in continuous measurement mode while participants viewed a high-contrast (96%) Maltese cross at both distance (4 m) and near (33 cm). Participants wore the study lenses in a trial frame while undergoing the procedure to record the measurements through either the CZ (unmasked lens) or PZ (masked lens), and were encouraged to keep the target in focus throughout the test duration. Accommodative responses were calculated based on the equations published by Atchison and Varnas [34]. Anomalous values (i.e., with a difference of more than 1.00 D between two consecutive readings or exceeding three standard deviations [35]) were excluded from the analysis. The standard deviation of the remaining data was calculated as the variability of the accommodative response within the measurement time [36].

### Statistical Analysis

Statistical analysis was performed with SPSS software (version 29.0, IBM, ibm.com/products/spss-statistics). Based on the Shapiro–Wilk test results, repeated-measure analysis of variance (ANOVA) was used to evaluate inter-lens differences. For data found to be significantly different from a normal distribution, the nonparametric Friedman test was used to compare inter-lens differences [37]. Where overall tests of differences results were found significant, either post-hoc or the Wilcoxon signed-rank test, pairwise comparisons were performed. All the presented *p*-values <0.05 were considered significant, with Bonferroni correction applied for pairwise comparisons.

## Results

Twenty participants (16 female) were recruited, with a mean (SD) age of 22.4 (±2.72) years and MSE −2.21 (±1.10), −0.40 (±0.24) D astigmatism and best-corrected VA of −0.05 (±0.07) logMAR.

### High Contrast VA

**Fig. 1:**
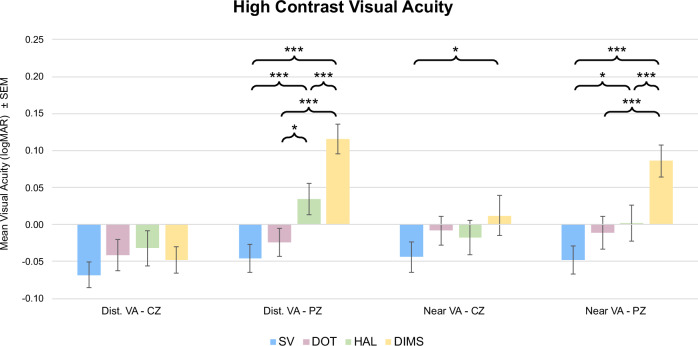
LogMAR high contrast visual acuity (VA) measured through the central zone (CZ) and peripheral zone (PZ) of standard single vision (SV), diffusion optics technology (DOT), highly aspheric lenslets (HAL) and defocus incorporated multiple segment (DIMS) lenses at distance (Dist., 4 m) and near (40 cm). Data presented as means, with error bars representing the standard error of the mean. Asterisks indicate significant differences between lenses (*p < 0.05, **p < 0.01 and ***p < 0.001).

A significant difference was found in high contrast VA between the four lenses while looking through the CZ at near (*p* = 0.005), but not at distance (*p* = 0.06) (Fig. 1); the significant difference was a reduction by 0.06 (±0.02) logMAR with DIMS compared to SV lenses (*p* = 0.04). Significant differences were found in high-contrast VA between the four lenses during both distance and near viewing (both *p* <0.001) while looking through the PZ (Fig. 1).

A significant reduction in high contrast distance VA was found with DIMS compared to SV (*p* <0.001), DOT (*p* <0.001) and HAL lenses (*p* = 0.02), consistent with a reduction in near high contrast VA compared to all other test lenses (all *p* <0.001). The high-contrast VA measured through the PZ was reduced by 0.16 (±0.02) logMAR at distance and 0.13 (±0.01) logMAR at near with DIMS compared to SV lenses.

HAL lenses also showed reduced high contrast VA through the PZ for both distance (*p* = 0.002) and near (*p* = 0.047) when compared to SV lenses, with mean differences of 0.08 (±0.02) logMAR at distance and 0.05 (±0.02) logMAR at near. In addition, HAL lenses exhibited a reduction in high-contrast distance VA through the PZ compared to DOT lenses (*p* = 0.04), whereas DOT lenses showed no significant impact on high-contrast VA at either distance (*p* = 0.85) or near (*p* = 0.09), compared with SV lenses.

### Low Contrast VA

**Fig. 2:**
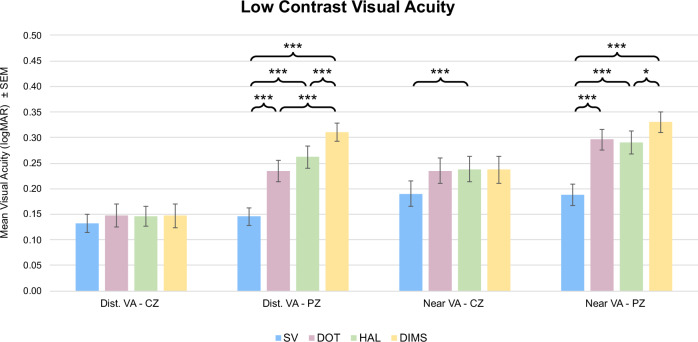
LogMAR low contrast visual acuity (VA) measured through the central zone (CZ) and peripheral zone (PZ) of standard single vision (SV), diffusion optics technology (DOT), highly aspheric lenslets (HAL) and defocus incorporated multiple segment (DIMS) lenses at distance (Dist., 4 m) and near (40 cm). Data presented as means, with error bars representing the standard error of the mean. Asterisks indicate significant differences between lenses (*p < 0.05, **p < 0.01 and ***p < 0.001).

A significant difference was found when comparing the low contrast VA assessed through the CZ of the four test lenses (*p* = 0.01), with a mean reduction of 0.05 (±0.01) logMAR in near VA between HAL and SV lenses (*p* = 0.02), but no significant difference at distance (*p* = 0.74) (Fig. 2).

Significant differences occurred between the four lenses for both distance and near low contrast VA while looking through the PZ (both *p* <0.001), with all three myopia control lens designs exhibiting reduced VA compared to SV lenses (all *p* <0.001) (Fig. 2).

Compared with SV lenses, mean reduced low contrast VA was found, with differences of 0.09 (±0.02), 0.12 (±0.02) and 0.17 (±0.02) logMAR at distance and 0.11 (±0.02), 0.10 (±0.02) and 0.14 (±0.01) logMAR at near with the DOT, HAL and DIMS lenses, respectively.

Among the three myopia control lenses, DIMS lenses showed reduced low-contrast VA compared to DOT (*p* <0.001) and HAL lenses (*p* = 0.02) at distance, while a significant difference at near was found in comparison to HAL lenses only (*p* = 0.02). No significant differences in low-contrast VA were found between DOT and HAL lenses at both distance and near when looking through the PZ.

### Contrast Sensitivity Function

**Fig. 3:**
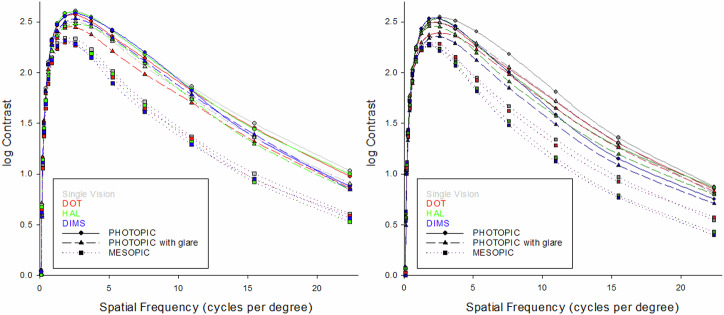
Contrast sensitivity function. Contrast sensitivity with spatial frequency function when viewing through the central zone (CZ) (left) and peripheral zone (PZ) (right) in photopic, photopic with glare and mesopic conditions for single vision (SV), diffusion optics technology (DOT), highly aspheric lenslets (HAL) and defocus incorporated multiple segment (DIMS) lenses.

Contrast sensitivity assessed under photopic conditions, without a glare source, showed no significant differences in performance across the test lenses, whether measured through the CZ (*p* = 0.80) or PZ (*p* = 0.06). However, contrast sensitivity was markedly lower when viewing through the PZ compared to the CZ (*p* <0.001), under both glare conditions and reduced mesopic luminance (for both, *p* <0.001). The introduction of a glare source (*p* = 0.02) or lowering luminance from photopic to mesopic levels (*p* <0.001) resulted in a reduction of contrast sensitivity. No significant differences were observed between the lenses under glare conditions (*p* = 0.06), but reducing the lighting to mesopic levels revealed significant differences (*p* = 0.005). In the CZ, all of the myopia control lenses did not perform as well as SV lenses (*p* <0.001), whereas in the PZ, DOT lenses performed similarly to SV lenses (*p* = 0.54), while HAL and DIMS lenses performed worse than both (*p* <0.01) (Fig. 3).

### Reading Performance Through PZ

**Fig. 4:**
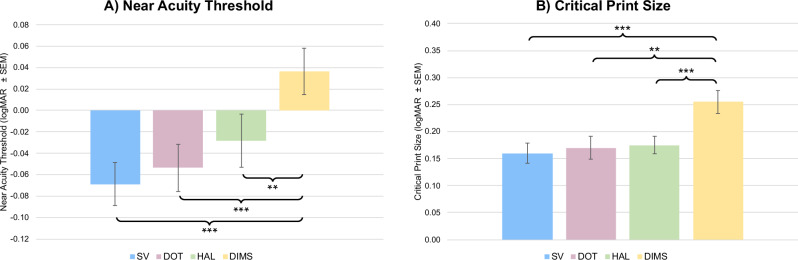
Near acuity threshold (**A**) and critical print size (**B**) with the single vision (SV), diffusion optics technology (DOT), highly aspheric lenslets (HAL) and defocus incorporated multiple segment (DIMS) lenses through the peripheral zone (PZ). Data presented as means, with error bars representing the standard error of the mean. Asterisks indicate significant differences between lenses (*p <0.05, **p < 0.01 and ***p < 0.001).

The mean maximum reading speeds through the PZ for SV (211.43 ± 39.30 words per minute), DOT (210.24 ± 37.00), HAL (209.22 ± 40.21) and DIMS (210.59 ± 39.08) lenses were similar (*p* = 0.94). Viewing through the PZ of the DIMS lens reduced the near acuity threshold compared to SV (*p* <0.001), DOT (*p* <0.001) and HAL (*p* = 0.008) lenses. DOT and HAL lenses showed no significant impact on the near acuity threshold when compared to SV lenses (*p* > 0.99 and *p* = 0.27, respectively) and there was no significant difference between DOT and HAL lenses (*p* = 0.99) (Fig. 4A). In addition, DIMS lenses resulted in a larger critical print size than SV (*p* <0.001), DOT (*p* = 0.003) and HAL lenses (*p* <0.001), while there were no significant differences between SV and DOT (*p* = 0.71) or HAL (*p* = 0.37), or between DOT and HAL lenses (*p* = 0.76) (Fig. 4B).

### Accommodative Facility

No significant difference was found in the number of errors recorded during the assessment of accommodative facility, alternating between CZ and PZ for the four lenses tested (*p* = 0.53) (Table 1). Although a significant difference was observed between the lenses in terms of the number of cycles recorded in 1 min (*p* = 0.003), no pairwise comparisons were significant (all *p* ≥ 0.14).

**Table 1:** Participants’ accommodative facility alternating between the central zone (CZ) and peripheral zone (PZ) and relative errors with the single vision (SV), diffusion optics technology (DOT), highly aspheric lenslets (HAL) and defocus incorporated multiple segment (DIMS) lenses.

	SV	DOT	HAL	DIMS
Number of focusing cycles per minute (interquartile range)	25.25 (23.75–27.50)	25.00 (23.50–29.75)	24.00 (22.5–27.38)	23.75 (21.50–26.50)
Number of Errors (interquartile range)	0.5 (0–2)	0.5 (0–1)	0.5 (0–2)	1 (0–1.75)

Data presented as median (interquartile range).

### Visual Search Task

The duration when performing the visual search task did not differ significantly between the lenses through both the CZ (*p* = 0.68) and PZ (*p* = 0.35) (Table 2). No significant difference in the number of errors was seen between the four lenses through the CZ (*p* = 0.40). However, significant differences were noted through the PZ (*p* = 0.048), due to fewer errors with the DOT compared with SV lenses (*p* = 0.005).

**Table 2:** Duration and the number of errors when performing the visual search tasks through the central zone (CZ) and peripheral zone (PZ) of the single vision (SV), diffusion optics technology (DOT), highly aspheric lenslets (HAL) and defocus incorporated multiple segment (DIMS) lenses.

Lens zone	SV	DOT	HAL	DIMS	Friedman test
Duration (s)					
CZ	38.36 (29.22–45.28)	36.45 (29.59–47.75)	38.62 (26.60– 49.04)	36.27 (28.20–44.80)	*p* = 0.68
PZ	35.56 (25.15– 48.87)	35.14 (27.81– 41.72)	39.70 (32.20– 46.38)	37.87 (30.97– 45.83)	*p* = 0.35
No. of errors					
CZ	0.00 (0–1)	1.00 (0–1.75)	1.00 (0–2)	1.00 (0–1)	*p* = 0.40
PZ	1.00 (0–2)	1.00 (0–1)	0.00 (0–1.75)	1.00 (0–1.75)	*p* = 0.048^*^

Data presented as median (interquartile range). Asterisks indicate significant differences between lenses (*p < 0.05).

### Accommodative Response and Variability

No significant differences in accommodative response were found between the lenses when measured through the CZ at far (*p* = 0.34). However, a significant difference was observed at near (*p* = 0.006), with the DOT lenses showing a lower response compared with DIMS lenses (*p* = 0.03), but not significantly different compared with SV (*p* = 0.07); nor were any other pairwise comparisons significant. The accommodative responses did not differ significantly between the lenses through the PZ at far (*p* = 0.13) or near (*p* = 0.32) (Table 3).

**Table 3:** Accommodative response through the single vision (SV), diffusion optics technology (DOT), highly aspheric lenslets (HAL) and defocus incorporated multiple segment (DIMS) lenses measured through the central zone (CZ) and peripheral zone (PZ) when fixating at far and near targets.

Lens zone	Accommodative response (D)	SV	DOT	HAL	DIMS	Repeated measures ANOVA
CZ	Far (0.25 D accommodative stimulus)	0.11 (0.28)	0.03 (0.24)	0.16 (0.56)	0.13 (0.32)	*p* = 0.34
	Near (3.00 D accommodative stimulus)	2.27 (0.26)	2.12^a^ (0.29)	2.31 (0.32)	2.37 (0.32)	*p* = 0.006
PZ	Far (0.25 D)	0.13 (0.30)	0.06 (0.32)	0.21 (0.49)	0.10 (0.30)	*p* = 0.13
	Near (3.00 D)	2.27 (0.30)	2.22 (0.23)	2.35 (0.40)	2.27 (0.37)	*p* = 0.32

Data are presented as mean (SD).

^a^Sig difference in pairwise comparison with DIMS lens (# *p* < 0.05).

**Fig. 5:**
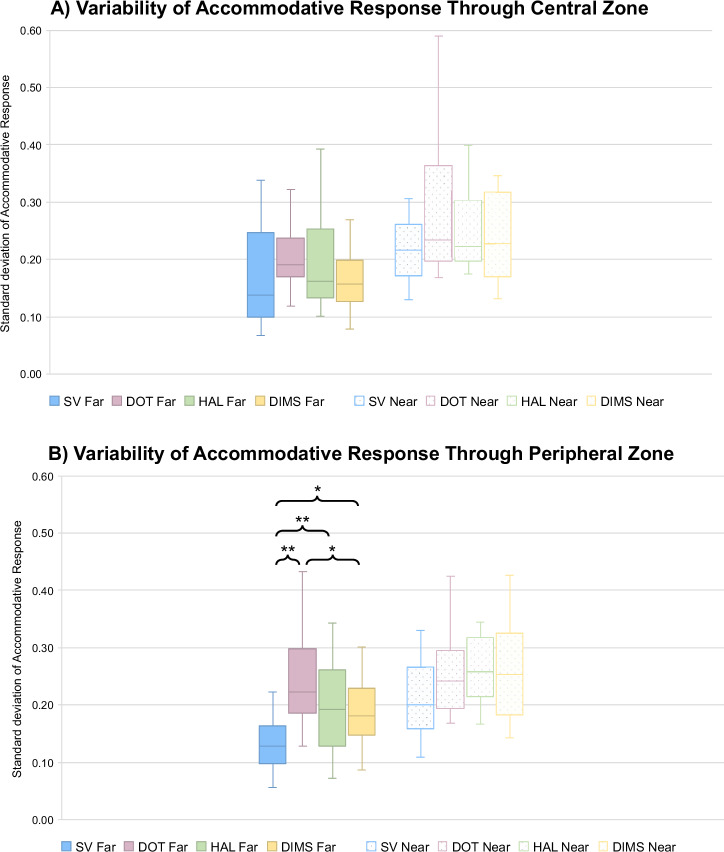
Accommodative response variability. Box plots showing variation of accommodative response through the central zone (**A**) and peripheral zone (**B**) of the single vision (SV), diffusion optics technology (DOT), highly aspheric lenslets (HAL) and defocus incorporated multiple segment (DIMS) lenses while looking at far and near targets. The line within the box is the median value, the box extents represent the quartiles, and the whiskers represent the maximum and minimum values. Asterisks indicate a significant difference between lenses (*p < 0.05, and **p < 0.01).

No significant differences in accommodative variability were found between the lenses through the CZ, with no pairwise comparisons reaching significance at far (after adjustment, for all pairwise *p* ≥ 0.17) or at near (*p* = 0.16) (Fig. 5A).

Far through the PZ, a significant difference was observed (*p* <0.001), with SV showing lower variability (Median 0.13D, IQR: 0.06D) than DOT (0.22D, IQR 0.12D, *p* <0.01), HAL (0.19D, IQR 0.13 D, *p* <0.01) and DIMS (0.18 D, IQR 0.08D, *p* = 0.04). DOT lenses also differed significantly from the DIMS lenses (*p* = 0.01) for the same condition. Near, accommodative variability did not differ significantly between lenses through either the CZ or the PZ (all *p* > 0.05) (Fig. 5B).

## Discussion

Myopia control spectacle lenses are gaining increasing popularity due to their high efficacy and safety. Simultaneously, there is increasing interest in how these lenses influence visual function and performance. As the variety of lens designs for myopia control continues to grow, more information is emerging about their effects on visual functions. This study is the first to evaluate contextually the visual performance of contrast modulation spectacle lenses and those based on defocus for myopia control.

The assessment of VA is a key factor in determining visual performance, and it has been studied extensively in the context of myopia control lenses, considering various conditions such as contrast, lighting and lens sectors [12]. Comparisons of VA across different myopia control lens designs have shown distinct visual performance outcomes, particularly when vision is assessed through the lens periphery.

In assessing VA through the lens CZ, no significant differences were found across the designs at distance for either high or low-contrast letters. At near distances, although the clinical relevance of the VA reductions remained marginal, significant differences were found for high and low contrast VA with DIMS and HAL lenses, respectively, aligning with previous reports on DOT [15], DIMS [14] and HAL [17] lenses. Although a positive effect of DIMS lenses, compared with SV, has also been reported after a 2-week adaptation period in young adults with high-contrast, but not for low-contrast letters, the differences were not deemed clinically significant [38].

Conversely, viewing through the PZ of myopia control lenses was associated with varying degrees of VA reduction across different lens designs, depending on the testing distance and letter contrast. Consistent with previous findings, use of the lens periphery in DIMS designs resulted in a significant reduction in VA compared with SV lenses at both distance and near, for both high and low-contrast targets [14, 19, 20]. Similarly, although less pronounced, assessing vision through the periphery of HAL lenses also showed a consistent reduction in VA compared with SV lenses across the conditions tested [17, 29]. Rather, DOT lenses demonstrated comparable results to SV lenses for high-contrast letters at both distance and near, while the reduction in low-contrast VA was similar to that induced by HAL and DIMS lenses. These differences may be partly explained by the distinct optical profiles of the lens designs. Defocus-modulation lenses incorporate discrete zones of positive power that generate superimposed focal planes and spatial-frequency dependent variations in contrast transmission [39–41], whereas the contrast-modulation design employs diffusive microstructures that modulate retinal image contrast more uniformly, producing a layered light distribution without distinct secondary foci [42–44]. Moreover, variations between defocus-based designs may also be related to the characteristics, arrangement and relative density of optical elements within the PZ, and the extent to which the retinal image is influenced by different lenslet architectures [18].

Therefore, vision in primary gaze appears to be preserved consistently, irrespective of the lens type. The CZ of myopia control lenses, whether designed for defocus or contrast modulation, can provide adequate retinal image quality to maintain best-corrected VA. Furthermore, potential optical interference from rays passing through the treatment zone in the paracentral portion of the lenses is expected to be negligible. This consideration is particularly relevant for DOT lenses, presenting a treatment zone extending to the near-peripheral retina, which has received growing interest due to its proposed role in the myopia control mechanism [45]. In addition, findings from VA assessments through the PZ suggest that visual outcomes can be influenced by the lens design, with additional variability being associated with the contrast level of the viewed target. Therefore, environmental and individual factors should be integrated into clinical discussions. While considerations regarding the contrast level of surrounding visual scenes fall beyond the scope of this study, previous research suggests that children’s visual exploration can be characterised by larger saccadic amplitudes than adults [46], thereby increasing the likelihood of foveal fixation through the PZ. However, head movement patterns have been reported to remain unaffected by the use of myopia control lenses, specifically those employing contrast modulation, thus supporting the notion that they can be well tolerated [15]. Therefore, while precise alignment of the CZ and optical centre in primary gaze is critical for optimising visual outcomes, children with a greater propensity for wider exploratory gaze behaviours may benefit from clinical guidance on the appropriate use of myopia control lenses, along with the selection of specific designs tailored to their visual habits.

Lighting conditions influenced the contrast sensitivity levels measured for the test lenses. While the presence of a glare source reduced contrast sensitivity equally, with no significant differences being observed across the lenses tested, the reductions observed at lower luminance levels and when assessing the PZ were dependent on lens design. Specifically, DOT lenses demonstrated contrast sensitivity comparable to SV lenses, whereas HAL and DIMS lenses exhibited a similar degree of contrast sensitivity deterioration relative to SV. Viewing through the CZ of any myopia control lens design resulted in an equivalent reduction in contrast sensitivity compared with SV lenses, although the magnitude of this reduction may not be clinically significant [15]. Reductions in contrast sensitivity with HAL and DIMS lenses have been reported, identifying luminance, spatial frequency and lens region as key contributing factors [18, 47, 48]. Interestingly, DOT lenses have been suggested to maintain comparable performance to SV lenses with both central and peripheral viewing [49, 50]. Nonetheless, it is worth noting that the methodology employed in this study enabled direct, continuous assessment of a contrast sensitivity curve across an extended range of spatial frequencies, whereas previous investigations varied in testing conditions, as well as in range and sampling density of spatial frequencies examined, constraining direct comparisons across studies. Hence, the findings of the present investigation corroborate the potential for reduced contrast sensitivity being associated with myopia control lenses, further suggesting that these designs may interfere with contrast detection as part of their mechanism of action [42, 51]. Nonetheless, considering that DOT lenses performed similarly to SV in PZ assessment, it appears that defocus modulation designs may exert a greater influence on contrast sensitivity than those specifically intended to modulate retinal image contrast [52], possibly reflecting the visual system’s ability to adapt to localised contrast reductions, thereby enhancing sensitivity [53].

The assessment of visual performance beyond VA and contrast sensitivity aimed to explore further visual aspects that may be influenced by the use of myopia control spectacle lenses. The selection of testing methodologies was guided by their potential representation of the dynamic use of spectacles and the ability to resemble daily activities, particularly at near distances.

Reading performance was evaluated using an extended set of MNRead test sentences, presented in digital format [28, 31]. The test was conducted through the PZ of the lenses to simulate reading in downgaze, as—despite some temporal variability—a consistently inferiorly projected angle of gaze during near activities has been identified [54]. Results indicated that while reading speed remained consistent across all test and control lenses, a significant difference in near acuity threshold was observed with the DIMS lenses. This finding partially aligns with previous reports on HAL lenses, which demonstrated reduced reading speed for low-contrast words when compared with SV lenses [29], suggesting that defocus modulation may introduce a minor, yet detectable, decline in near visual performance. However, the observed differences may fall within a range comparable to the inherent variation in critical print size and fluent reading range [55], which could also explain why significant differences were not identified for both lens designs. Thus, the retinal image quality produced by the PZ of all myopia control lens designs appears sufficient to prevent any detrimental impact on near reading performance, while the contrast modulation design may enhance letter detail discrimination in a manner comparable to SV lenses [15], as observed in the high contrast near VA outcomes.

Accommodative facility and visual search tasks can offer potential insights to deepen understanding of the visual performance associated with different lens designs. All myopia control designs demonstrated a consistent level of focusing performance comparable with SV lenses—as assessed by the Hart Chart test, in line with results reported for defocus modulating designs using a ±2.00 D lens flipper [38, 56]. Notably, the contrast-modulation design appeared less prone to induce errors in the visual search through the PZ compared with SV lenses, an aspect not previously explored with myopia control lenses [12]. Although no other significant differences were observed between the defocus-modulation designs and SV lenses, or between the contrast- and defocus-modulation designs, these findings may provide a broader context for interpreting outcomes in VA and reading performance, suggesting that DOT lenses may exert less interference on letter/symbol recognition [12].

This study provides the first direct comparative assessment of objectively evaluated accommodative behaviour between defocus and contrast modulation lenses, investigating their influence on accommodative response and its variability through the CZ and PZs of the myopia control spectacle lenses in foveal monocular measurements. No significant differences in accommodative response emerged between myopia control and SV lenses, either when assessed through the central or the PZ at both distance and near, with the only significant difference found through the CZ, with a slightly reduced amount of accommodation elicited by DOT compared with DIMS lenses when fixating a near target. These results were in line with previous findings on both defocus [9, 10, 14, 56–58] and contrast modulation lenses [16], where the accommodative response was found to be comparable with SV lenses. Accommodative variability was generally consistent across lenses. A modest increase in variability was observed only at distance fixation through the PZ, where SV lenses showed lower variability than the myopia control designs and a minor difference between DOT and DIMS lenses was also observed, in line with evidence for increased accommodative variability for defocus modulating lenses [56, 57].

Interpretation of objectively assessed accommodative data requires particular caution, as numerous factors can influence the outcomes. Differences in study protocols, such as the choice of measurement targets, viewing conditions and adaptation periods, as well as variations in instruments and their setup, can contribute to variability across studies [59, 60]. The use of contact lenses to compensate for refractive error should not affect the evaluation of accommodation [61], whereas assessments conducted through spectacle lenses with complex designs may influence the results [62], with differences depending on whether accommodation is measured directly through the spectacle lens or by consensual response [38, 60]. A possible link may reside in the interaction between the optical properties of myopia-control lens designs and the instruments employed for detection. Infrared autorefractors have been widely used to assess the accommodative response, representing a reliable and valid tool for both optometric practice [25] and myopia research [63]. Nonetheless, their spatial [64] and temporal [25] resolution may limit the investigation of accommodative behaviour with complex lens designs. In particular, instruments that derive refraction from retinal reflections of target ring [64], may be challenged by scattering and multiple focal signals introduced by myopia control lenses for wavefront analysis [39, 40]. The interaction between lens design and autorefractor measurement principles may influence how the reflected infrared beam is processed and averaged, potentially introducing variability or artefacts in the accommodative response data. Consistent with the considerations on optics and VA, interpretation of objective accommodative outcomes with complex myopia control lenses should be approached cautiously, bearing in mind that defocus-modulating lenses have been associated with spatial-frequency-dependent changes in contrast transmission, whereas contrast-modulating lenses tend to exert a more uniform effect across spatial frequencies.

However, despite the complexity of this optical and physiological framework, subjective assessments of lens performance have shown that myopia control designs, while differing between defocus and contrast modulation, can offer visual outcomes comparable to those of SV lenses, as also observed here. This further suggests that the visual system possesses considerable capacity to adapt to the optical modifications introduced by these lenses, enabling satisfactory visual function even in the presence of altered retinal image quality or contrast profiles [44, 65, 66]. Therefore, it should be recognised that subjective outcomes may not reflect fully the underlying optical differences or their objective measurements, and individuals can report satisfactory visual quality even when measurable alterations in optical performance are present. While objective measurements can be important for a comprehensive understanding of the visual impact of myopia control lenses, subjective assessment outcomes should be considered the most relevant indications of real-world visual experience with myopia management lenses.

Further research is suggested to validate these findings in a cohort of myopic children, particularly considering that the experimental design adopted in this study—assessing the immediate effects of myopia control lens designs—differed from those previously employed in younger wearers with adaptation periods, lens designs investigated and partially testing procedures. However, it should be considered that the complexity of certain testing protocols may discourage the use of extensive test batteries with children [67], despite their comfort and visual demands being similar to those of young adults [68]. Nonetheless, the validity of the outcomes presented in this study can be supported by evidence indicating that visual performance with myopia control spectacle lenses may be comparable between children and adults [18, 29], and that young adults may be even more susceptible to, or aware of, visual performance deterioration [69]. Furthermore, conducting a complex protocol in young adults provides a foundation for identifying the most relevant and clinically applicable tests for evaluating visual performance and requirements in children.

In addition, the setup of both participants and lenses in the visual performance assessments may impose limitations on the broader applicability of the findings. The experimental setup allowed for a comprehensive evaluation of visual performance in primary gaze and confirmed that myopia control lenses perform equivalently to SV lenses through their central areas. This should highlight further the importance of ensuring that accurate frame fitting is conducted to achieve stable and comfortable alignment of the central optical zone, or ideally the optical centre, in accordance with the wearer’s characteristics. Conversely, assessment through the PZ was employed primarily to ensure a consistent and repeatable evaluation of corresponding areas of the lenses. Accordingly, the masking of the lenses and the requirement for headrest-based testing, implemented to assess equivalent PZ at an equal distance from the optical centre across designs, restricted any positional adaptations that could mitigate potential interferences of the treatment zones on visual performance. Nonetheless, as compensatory head movements have been reported to remain unchanged between SV and myopia control lenses, specifically, those with contrast modulation design [15], further research is necessary to assess whether head posture adaptations may influence the testing outcomes reported.

## Conclusions

All three myopia control lenses performed well against an SV comparator through both the lens centre and periphery. The results suggest wearers’ vision should not be impaired significantly, irrespective of the lens design. However, clinicians should be cognisant of potential differences in visual performances, particularly through the lens periphery, between defocus and contrast-modulating designs. Nevertheless, attention to fitting, ensuring optimum horizontal and vertical centration and to the visual requirements when discussing treatment options is likely to maximise visual performance and comfort with myopia control lenses.

## Data Availability

Dataset and/ or detailed analysis outputs are available upon reasonable request to the corresponding author.
